# Role of ADAMTS13, VWF and F8 genes in deep vein thrombosis

**DOI:** 10.1371/journal.pone.0258675

**Published:** 2021-10-18

**Authors:** Maria Teresa Pagliari, Andrea Cairo, Marco Boscarino, Ilaria Mancini, Emanuela Pappalardo, Paolo Bucciarelli, Ida Martinelli, Frits R. Rosendaal, Flora Peyvandi

**Affiliations:** 1 Fondazione IRCCS Ca’ Granda Ospedale Maggiore Policlinico, Angelo Bianchi Bonomi Hemophilia and Thrombosis Center, Milan, Italy; 2 Department of Pathophysiology and Transplantation, Università degli Studi di Milano, Milan, Italy; 3 Department of Clinical Epidemiology, Leiden University Medical Center, Leiden, Netherlands; National Cerebral and Cardiovascular Center, JAPAN

## Abstract

**Background:**

We previously described the association between rare *ADAMTS13* single nucleotide variants (SNVs) and deep vein thrombosis (DVT). Moreover, DVT patients with at least one rare *ADAMTS13* SNV had a lower ADAMTS13 activity than non-carriers.

**Aims:**

To confirm *ADAMTS13* variants association with DVT and reduced plasma ADAMTS13 activity levels in a larger population. To investigate the role of *VWF* and *F8* variants.

**Methods:**

*ADAMTS13*, *VWF* and *F8* were sequenced using next-generation sequencing in 594 Italian DVT patients and 571 controls. Genetic association testing was performed using logistic regression and gene-based tests. The association between rare *ADAMTS13* variants and the respective plasmatic activity, available for 365 cases and 292 controls, was determined using linear regression. All analyses were age-, sex- adjusted.

**Results:**

We identified 48 low-frequency/common and 272 rare variants. Nine low-frequency/common variants had a P<0.05, but a false discovery rate between 0.06 and 0.24. Of them, 7 were found in *ADAMTS13* (rs28641026, rs28503257, rs685523, rs3124768, rs3118667, rs739469, rs3124767; all protective) and 2 in *VWF* (rs1800382 [risk], rs7962217 [protective]). Rare *ADAMTS13* variants were significantly associated with DVT using the burden, variable threshold (VT) and UNIQ (P<0.05), but not with C-ALPHA, SKAT and SKAT-O tests. Rare *VWF* and *F8* variants were not associated with DVT. Carriers of rare *ADAMTS13* variants had lower ADAMTS13 activity than non-carriers (ß -6.2, 95%CI -11,-1.5). This association was stronger for DVT patients than controls (ß -7.5, 95%CI -13.5,-1.5 vs. ß -2.9, 95%CI -10.4,4.5).

**Conclusions:**

*ADAMTS13* and *VWF* low-frequency/common variants mainly showed a protective effect, although their association with DVT was not confirmed. DVT patients carrying a rare *ADAMTS13* variants had slightly reduced ADAMTS13 activity levels, but a higher DVT risk. Rare *VWF* and *FVIII* variants were not associated with DVT suggesting that other mechanisms are responsible for the high VWF and FVIII levels measured in DVT patients.

## Introduction

Deep vein thrombosis (DVT) is a common life-threatening thrombotic disorder caused by the shift of the hemostatic equilibrium toward the blood clot formation. DVT along with pulmonary thromboembolism constitute venous thromboembolism (VTE), which is characterized by an annual incidence of 2–3 per 1000 individuals [1]. Part of DVT events is explained by a strong genetic component that includes the deficiencies of natural anticoagulants proteins (antithrombin, protein C and protein S), factor V Leiden (FVL), prothrombin G20210A mutation, fibrinogen gamma chain C10034T mutation [2]. The dissemination and improvement of new sequencing approaches including genome-wide association studies (GWAS) along with meta-analyses of GWAS data, led to the identification of new loci which mainly refer to genes involved in the hemostatic pathways [3, 4]. However, these genetic risk factors contribute to explain only 50–60% of DVT genetic hereditability [5–7]. Our group previously reported the results obtained using DNA next-generation sequencing (NGS) to evaluate the role of the disintegrin and metalloprotease with thrombospondin type 1 motif, number 13 (ADAMTS13) and von Willebrand factor (VWF) genes which encode two proteins involved in the maintenance of the equilibrium between hemostasis and thrombosis. In particular, we showed that DVT patients had an excess of rare *ADAMTS13* single nucleotide variants (SNVs) compared with controls. Moreover, DVT patients carrying at least one rare SNV showed a lower ADAMTS13 activity than non-carrier patients [8]. The functional effect of these variants has been evaluated by performing *in vitro* expression studies, which confirmed a reduction of ADAMTS13 activity for 3 out of 9 variants (p.V154I, p.D187H and p.R421C) [9].

Based on this background, we aimed to confirm our previous results which suggested an association between rare *ADAMTS13* variants and DVT (odds ratio [OR] 4.8; 95% confidence interval [CI] 1.6–15.0) [8]. To this purpose, a total of 594 Italian DVT patients and 571 controls were sequenced using NGS. Then, the association between carrier-ship of a rare *ADAMTS13* variant and ADAMTS13 activity levels was evaluated. The study also focused on *VWF* and *F8* due to the strong role of the respective encoded proteins in the maintenance of hemostasis and thrombosis equilibrium and because increased VWF and FVIII levels have been already described as associated with an increased DVT risk [10].

## Materials and methods

### Study population

We selected Italian DVT patients from those referred to the Angelo Bianchi Bonomi Hemophilia and Thrombosis Center in Milan (Italy) between 2006 and 2016. Briefly, the selection criteria included: (i) objective diagnosis of DVT of the lower limbs (i.e., by compression ultrasonography or venography); (ii) idiopathic DVT defined by the absence of cancer, surgery or immobilization; (iii) normal levels of the natural anticoagulant proteins antithrombin, protein C and protein S; (iv) absence of FVL or prothrombin G20210A mutations. Controls, matched with cases for age (+/- 5 years) and sex were recruited among friends and partners who accompanied patients to the Center, agreed to be tested for thrombophilia and had wild-type FVL and prothrombin G20210A genotypes [9].

ADAMTS13 activity, previously measured using the FRET-VWF73 assay [11], was available in 365 DVT patients and 292 controls [12]. The study was approved by the Ethics Committee of the Fondazione IRCCS Ca’ Granda Ospedale Maggiore Policlinico. All study subjects were aware of the content of this study and gave a written informed consent.

### Next generation sequencing, quality control and data analyses

NGS experiments were performed in two stages. For 298 DVT cases and 298 controls, the coding region, intron-exon boundaries, and 3’ and 5’ untranslated regions (UTRs) of *ADAMTS13*, *VWF* and *F8* were sequenced as part of a larger panel as previously reported [13]. Briefly, a multiplexed NGS (Human Genome Sequencing Center, Baylor College of Medicine, Houston, USA) was performed using unique barcode-sequencing tags, to create library pools of 8–20 samples, which were captured and sequenced in parallel using the Illumina HiSeq 2000 sequencing platform (Illumina, San Diego, USA). Reads were mapped to reference genome GRCh37/hg19 by use of the Burrows–Wheeler aligner (BWA)-MEM, resulting in BAM files per sample [13].

A second NGS panel (Illumina, San Diego, USA) limited to the coding region, intron-exon boundaries, and 3’ and 5’ untranslated regions (UTRs) of *ADAMTS13*, *F8* and *VWF* was subsequently designed to increase our study population by sequencing additional 296 cases and 273 controls. The sequences were aligned to the same reference genome (GRCh37/hg19) using the BWA-MEM algorithm. Picard and GATK tools were applied to sort index and recalibrate the BAM files of the individual subjects. Then, BAM files obtained from both sequencing panels underwent variant calling, performed using Haplotype Caller (GATK). The single gVCF files were assembled in a unique file and the variants were filtered according to the guidelines reported by the Broad Institute (https://software.broadinstitute.org/gatk/best-practices/). Variants’ annotation was performed using wANNOVAR (http://wannovar.wglab.org/). A further quality control was performed using KGGSeq (http://grass.cgs.hku.hk/limx/kggseq/), excluding variants with an average reading depth < 10, Phred score < Q30 and Hardy-Weinberg equilibrium P < 1.0 E-04. Variants were divided on the basis of their minor allele frequency (MAF) in low-frequency/common (MAF ≥ 1%) and rare (MAF < 1%).

### Statistical analysis

Continuous variables were described as median and interquartile range (IQR), whereas categorical variables were reported as counts and percentages. ADAMTS13 activity, von Willebrand factor antigen (VWF:Ag) and factor VIII coagulant activity (FVIII:C) levels in DVT patients and controls were compared using the non-parametric Mann-Whitney test. P-values < 0.05 were considered statistically significant. Low-frequency and common variants were individually analyzed using a logistic regression model age- and sex- adjusted and considering a model of additive inheritance with PLINK (http://zzz.bwh.harvard.edu/plink/contact.shtml). Results were reported as OR with the corresponding 95% CI. The False discovery rate (FDR) was used to correct for multiple testing and variants with an FDR < 0.25 were further considered. Rare variants were analyzed using seven different cumulative association tests based on different analytical approaches: (i) unidirectional tests which include burden [14], variable threshold (VT [15]), UNIQ [16] and SUMSTAT tests [17]; (ii) bi-directional variance-component tests which allow different variants effects (neutral/deleterious) such as C-ALPHA [18] and the Sequence Kernel Association Test (SKAT [19]); and (iii) a combination of both unidirectional and bi-directional variance-component tests, represented by SKAT-O [20]. All gene-based association tests were performed with the PLINK/SEQ suite (https://atgu.mgh.harvard.edu/plinkseq/) and adjusted for age and sex [14].

The association between rare *ADAMTS13* variants and ADAMTS13 activity levels was evaluated using linear regression models adjusted for age and sex, by considering: (i) all variants identified in *ADAMTS13*; (ii) the variants potentially affecting ADAMTS13 activity such as missense mutations, frameshift mutations, deletions and insertions; (iii) the variants potentially affecting ADAMTS13 activity also predicted as damaging by CADD algorithm (score > 20) [21]. The analyses were performed in all individuals with an available measurement of ADAMTS13 activity, then stratifying by case-control status. Results were reported as beta coefficients with 95% CI. Statistical analyses were performed using the statistical software R, release 3.3.2 (R Foundation for Statistical Computing, Vienna, Austria).

## Results

Baseline characteristics of the study population, which include 594 cases and 571 controls, are reported in Table 1. About 60% of DVT patients were women with a mean age of 47 years, similar to controls. DVT patients showed higher median VWF:Ag (169% vs. 115%; P < 0.0001) and FVIII:C (148% vs. 113%, P < 0.0001) plasma levels than controls (Table 1). ADAMTS13 activity, available in 365 DVT patients and 292 controls, was slightly reduced in DVT patients than controls (94% vs. 98%, respectively; P = 0.02) [12].

**Table 1:** Baseline characteristics of the study population.

	DVT Patients	Controls	P *
N	594	571	-
Age (years), mean (SD)	47 ± 15	45 ± 14	-
Female sex, n (%)	328 (57)	313 (55)	
ADAMTS13 activity (%), median (IQR)^a^	94 (81–108)	98 (86–112)	P = 0.02
VWF antigen, median (IQR)^b^	169 (136–209)	115 (87–148)	P < 0.0001
FVIII coagulant activity, median (IQR)^c^	148 (124–173)	113 (93–137)	P < 0.0001
Time from acute event (months), median (IQR)	9 (3–32)	-	-
Patients treated at sampling time, n (%)^d^	174 (48)	-	-

^a^ Available in 365 cases and 292 Controls.

^b^ Available in 342 cases and 247 controls.

^c^ Available in 330 cases and 292 Controls.

^d^ Warfarin (n = 81), low molecular weight heparin (n = 54), Rivaroxaban (n = 25), unfractionated heparin (n = 6), Fondaparinux (n = 5), Apixaban (n = 1).

* Mann-Whitney test was performed. P values < 0.05 were considered statistically significant.

NGS identified a total of 320 variants distributed in the *ADAMTS13*, *VWF* and *F8*. These SNVs were mainly localized in the coding regions (77.5%) and introns (16%), whereas only 6.5% was found in the UTRs of sequenced genes. Forty-eight low-frequency/common variants, all missense, were distributed as follows: 18 in *ADAMTS13*, 27 in the *VWF* and 3 in the *F8* (S1 Table). Of 272 rare variants (MAF < 1%), 210 were localized in exons and included 126 missense, 80 synonymous mutations, 1 in-frame insertion, 2 in-frame deletions and 1 frameshift mutation, whereas the remaining 62 variants were found in introns and UTRs. Seventy out of 272 variants were referred as *novel* because not present in dbSNP or other databases. The complete list of rare variants is reported in the S2 Table.

A total of 89 rare variants were found in *ADAMTS13* and spanned across the gene. Of them, 48 were found in DVT patients, 28 in controls and 13 were found in both. Of the remaining 183 rare variants, 149 were in *VWF* and 34 in *F8*. Rare *VWF* and *F8* variants were homogeneously distributed among cases and controls (93 vs. 105 for *VWF*; 17 vs. 20 for *F8*). A total of 3 tri-allelic variants were identified (n = 1 in *ADAMTS13* and n = 2 in *VWF*).

### Low-frequency/common variants

**Fig 1:**
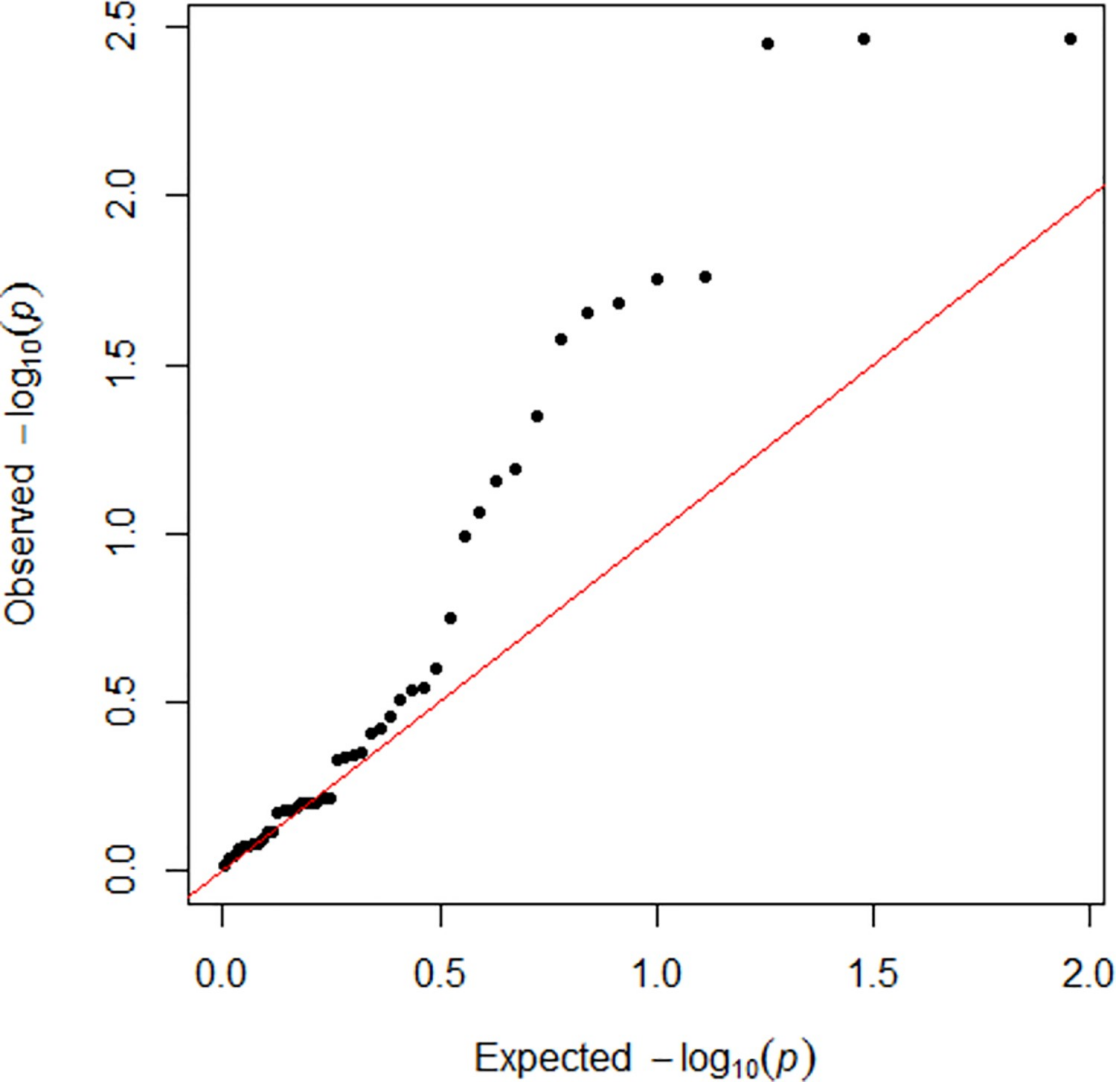
Quantile-quantile plot. P-value distributions referred to the 48 low-frequency/common variants (MAF ≥ 1%) identified in Italian DVT patients and matched controls sequenced by next-generation sequencing.

The QQ plot of the observed P-values vs. the expected distribution showed a signal inflation due to high the linkage disequilibrium of variants (Fig 1). Of 48 low-frequency/common SNVs, 9 showed a P < 0.05, but an FDR between 0.06 and 0.24 after correction for multiple testing (Table 2). Seven out of these 9 SNVs were located in *ADAMTS13* and two in the *VWF*. None of the 3 SNVs identified in *F8* were found to be associated with DVT. Low-frequency/common *ADAMTS13* variants showed a protective effect on DVT onset, with size effect estimates ranging from an OR 0.53 to 0.82. *ADAMTS13* SNVs rs3124768, rs3118667 and rs739469 showed the same OR of 0.78, explained by the high linkage disequilibrium among them (r^2^ between 0.74 and 1 for each combination). Similar results were found for *ADAMTS13* SNVs rs28641026 and rs28503257, which were associated to DVT with an OR of 0.53 (r^2^ = 1). *VWF* SNV rs1800382 had a strong association with DVT (OR 3.26, 95% CI 1.18–8.98; P = 0.02), whereas the rs7962217 had a protective effect on disease onset (Table 2).

**Table 2:** Low-frequency and common variants associated with DVT.

Gene	rsID	Chr	Position	Class	Ref. Allele/ Risk allele	Amino Acid Position	MAF Risk allele (Cases/ Controls)	OR (95%CI) ^a^	P	FDR
*ADAMTS13*	rs28641026	9	136314952	exonic	C/T	p.V970V	0.02/0.04	0.53 (0.31–0.89)	0.02	0.15
*ADAMTS13*	rs28503257	9	136319589	exonic	G/A	p.A1033T	0.02/0.04	0.53 (0.31–0.89)	0.02	0.15
*ADAMTS13*	rs685523	9	136310908	exonic	C/T	p.A900V	0.07/0.10	0.74 (0.55–0.99)	0.05	0.24
*ADAMTS13*	rs3124768	9	136304497	exonic	G/A	p.T572T	0.43/0.50	0.78 (0.67–0.92)	0.003	0.06
*ADAMTS13*	rs3118667	9	136291063	exonic	T/C	p.A140A	0.36/0.43	0.78 (0.66–0.92)	0.003	0.06
*ADAMTS13*	rs739469	9	136298729	intronic	C/G	.	0.43/0.49	0.78 (0.67–0.92)	0.004	0.06
*ADAMTS13*	rs3124767	9	136308542	exonic	T/C	p.G760G	0.45/0.50	0.82 (0.69–0.97)	0.02	0.15
*VWF*	rs1800382	12	6128388	exonic	C/T	p.R1399H	0.01/0.001	3.26 (1.18–8.98)	0.02	0.15
*VWF*	rs7962217	12	6061559	exonic	C/T	p.G2705R	0.05/0.07	0.67 (0.47–0.95)	0.03	0.16

Chr, chromosome; Ref. allele, Reference allele; MAF, Minor allele frequency; OR–odds ratio; 95% CI–confidence interval; FDR–false discovery rate; age/sex adjusted.

### Rare variants

The association between rare variants and DVT was assessed using different gene-based tests and results are summarized in Table 3. Because these tests consider the variant position, the three tri-allelic variants are not included in the count herein reported. *ADAMTS13* SNVs (n = 88) were found to be associated with DVT using the burden test (P = 0.02), VT (P = 0.03), and UNIQ (P = 0.04), but not with C-ALPHA, SUMSTAT, SKAT tests and SKAT-O (P > 0.05). Both burden and VT tests remained significant after performing a restriction analysis on potentially damaging variants (n = 45; P = 0.02 and P = 0.04, respectively). The same results were obtained by restricting the analysis to the 15 potentially damaging *ADAMTS13* variants also predicted to be damaging by CADD > 20 (burden, P = 0.003; VT, P = 0.005). SKAT-O showed a significant association after restriction analyses to potentially damaging variants (P = 0.05) and potentially damaging variants with CADD > 20 (P = 0.008).

**Table 3:** The association between *ADAMTS13*, *VWF* and *FVIII* rare variants and DVT assessed using different gene-based tests.

Rare variants					Potentially Damaging ^a^ mutations				CADD > 20			
Gene	N	Test	P	I	N	Test	P	I	N	Test	P	I
*ADAMTS13*	88 ^b^	BURDEN	0.02	0.001	45 ^b^	BURDEN	0.02	0.001	15	BURDEN	0.003	0.0003
		VT	0.03	0.003		VT	0.04	0.004		VT	0.005	0.0003
		UNIQ	0.04	0.003		UNIQ	0.08	-		UNIQ	0.09	0.006
		SUMSTAT	0.18	0.003		SUMSTAT	0.30	0.004		SUMSTAT	0.18	0.0003
		C-ALPHA	0.14	0.003		C-ALPHA	0.11	0.004		C-ALPHA	0.09	0.0003
		SKAT	0.19	-		SKAT	0.15	-		SKAT	0.09	-
		SKAT-O	0.06	-		SKAT-O	0.05	-		SKAT-O	0.008	-
*VWF*	147 ^b^	BURDEN	0.21	0.019	72 ^b^	BURDEN	0.31	0.032	35 ^b^	BURDEN	1	0.200
		VT	1	0.024		VT	0.64	0.008		VT	1	0.005
		UNIQ	1	0.200		UNIQ	1	0.200		UNIQ	1	0.200
		SUMSTAT	0.5	0.024		SUMSTAT	0.30	0.008		SUMSTAT	0.08	0.005
		C-ALPHA	0.26	0.024		C-ALPHA	0.10	0.008		C-ALPHA	0.06	0.005
		SKAT	0.13	-		SKAT	0.06	.		SKAT	0.02	-
		SKAT-O	0.23	-		SKAT-O	0.14	-		SKAT-O	0.04	-
*F8*	34	BURDEN	0.71	0.167	11	BURDEN	1	0.200	3	BURDEN	0.83	0.200
		VT	1	0.167		VT	1	0.005		VT	0.38	0.174
		UNIQ	0.7	0.167		UNIQ	1	0.200		UNIQ	0.83	0.200
		SUMSTAT	0.71	0.167		SUMSTAT	0.08	0.005		SUMSTAT	0.54	0.043
		C-ALPHA	0.86	0.167		C-ALPHA	0.06	0.010		C-ALPHA	0.46	0.130
		SKAT	0.64	-		SKAT	0.36	-		SKAT	0.35	-
		SKAT-O	0.81	-		SKAT-O	0.31	-		SKAT-O	0.47	-

P, P value based on permutation. I, proportion of null replicates for which the best test statistic was tied. All tests were adjusted for age and sex.

^a^ Potentially damaging mutations include missense mutations, frameshift mutations, deletions and insertions. N, number of variants analysed. VT, variable threshold.

^b^ Analyses have been done by considering variant position. Therefore, the tri-allelic variant identified in ADAMTS13 and VWF genes (n = 1 and n = 2, respectively) were not included in the count. The complete list of annotated variants is reported in the S2 Table.

None of these tests showed an association between rare *VWF* variants and DVT, neither considering all identified variants nor by performing a restriction analysis on potentially damaging variants. SKAT and SKAT-O showed a significant association after restriction analysis to *VWF* potentially damaging variants with a CADD > 20. Rare *F8* variants were not associated with DVT, even after restriction analyses (Table 3).

### Association between rare *ADAMTS13* SNVs and ADAMTS13 activity levels

This part of the study was performed by selecting sequenced cases and controls (n = 365 and n = 292, respectively) who had available ADAMTS13 activity levels, measured by the FRET-VWF73 assay. The association between the presence of at least one rare *ADAMTS13* variant and the related protein activity levels was assessed using linear regression adjusted for age and sex. The first analysis was performed by considering together patients and controls carriers for rare *ADAMTS13* variants (Table 4). Carriers of at least one rare variant (n = 71) showed reduced ADAMTS13 activity levels compared with non-carriers (n = 586; β -6.2, 95% CI -11, -1.5; P = 0.01). The association became stronger considering those variants potentially affecting ADAMTS13 activity (missense mutations, frameshift mutations, deletions and insertions [n = 33; β -11.0, 95% CI -18.0, -4.3; P = 0.001]) or variants potentially affecting ADAMTS13 activity with a CADD > 20 (n = 13; β -28, 95% CI -39.0, -18.0; P < 0.001). Secondly, we considered patients and controls, separately (Table 4). The association for patients was similar to that of the main analysis for each category of variants evaluated, with the highest association for those variants with CADD > 20 (n = 13; β -25.9, 95% CI -36, -16; P < 0.001). Conversely, no association was found in controls (n = 28; β -2.9, 95% CI -10.4, 4.5; P = 0.4).

**Table 4:** Association between rare *ADAMTS13* variants and ADAMTS13 activity levels in the whole population and after stratifying on case-control status.

	Carriers		Non-carriers		Linear Regression ^a^	
**Variant Class**	**All, n**	**ADAMTS13 Activity (%)**	**All, n**	**ADAMTS13 Activity (%)**	**ß (95% CI)**	**P**
*All rare*	71	91 (80–106)	586	96 (84–109)	-6.2 (-11.0, -1.5)	0.01
*Potentially damaging* ^b^	33	89 (71–100)	624	96 (84–109)	-11.0 (-18.0, -4.3)	<0.001
*CADD > 20*	13	63 (58–81)	644	96 (84–109)	-28.0 (-39.0, -18.0)	<0.001
**Variant Class**	**Cases, n**	**ADAMTS13 Activity (%)**	**Cases, n**	**ADAMTS13 Activity (%)**	**ß (95% CI)**	**P**
*All rare*	43	90 (72–98)	322	95 (81–108)	-7.5 (-13.0, -1.5)	0.014
*Potentially damaging* ^b^	24	82 (60–94)	341	94 (82–108)	-14.5 (-22.0, -6.8)	<0.001
*CADD > 20*	13	63 (58–81)	352	94 (82–108)	-25.9 (-36.0, -16.0)	<0.001
**Variant Class**	**Controls, n**	**ADAMTS13 Activity (%)**	**Controls, n**	**ADAMTS13 Activity (%)**	**ß (95% CI)**	**P**
*All rare*	28	98 (85–112)	264	98 (86–111)	-2.9 (-10.4, 4.5)	0.4
*Potentially damaging* ^b^	9	100 (86–114)	283	98 (86–111)	-2.4 (-10.0, 15.0)	0.7
*CADD > 20*	0	-	292	98 (86–111)	-	-

Analyses have been performed by considering at first all carriers of at least one rare ADAMTS13 variant and then by dividing carriers in DVT patients (cases) and controls. ADAMTS13 activity (expressed as percentage) was reported as median and interquartile range. Linear regression results were reported as beta coefficients (ß) and 95% confidence intervals (CI).

^a^ The model was adjusted for age and sex.

^b^ Potentially damaging mutations include missense mutations, frameshift mutations, deletions and insertions.

## Discussion

In the last years, the role of the *ADAMTS13* gene as a genetic risk factor for both arterial and venous thrombosis has been explored. Different authors reported the existing link between *ADAMTS13* variants and stroke or other cardiovascular diseases [22, 23], whereas our group was the first to describe an excess of rare *ADAMTS13* variants in a small group of 94 DVT patients and 98 controls. Furthermore, we showed that patients carrying at least one rare *ADAMTS13* variant had lower ADAMTS13 activity levels than non-carriers [8].

In the present study, we aimed to confirm our previous results in a larger population of 594 Italian DVT patients and 571 controls, focusing on ADAMTS13 along with VWF and F8 genes. We found a total of 320 variants which included 272 rare and 48 low-frequency/common variants mainly located in *ADAMTS13* and *VWF*, whereas those in *F8* were only a few. Similar to our previous study, DVT patients showed an excess of rare *ADAMTS13* variants than controls (61 vs. 41). These variants spanned across the gene, without highlight clusters [8]. A total of 15 rare SNVs were predicted as potentially damaging with CADD score > 20 (S2 and S4 Tables). Of them, 2 have already been identified in our previous studies and their effect on ADAMTS13 activity was confirmed by *in vitro* expression studies [9]. In addition, 1 DVT patient carrying the p.P1218C mutation showed an ADAMTS13 activity slightly below the normal range (42% vs. 45%).

The association between rare variants and DVT was tested using different cumulative association methods [14–20]. All rare *ADAMTS13* variants were associated with DVT using the burden and VT tests and the association was maintained even after restriction analyses to potentially damaging variants or potentially damaging variants with CADD > 20. The restriction analyses results were also confirmed by SKAT-O, a powerful method that combines the assumptions of unidirectional and bi-directional variance- component methods. Differently, UNIQ showed a significant association only when used to test all rare *ADAMTS13* variants, whereas SUMSTAT never showed any association. These inconsistencies can be related to the limits of the tests used. Indeed, both UNIQ and SUMSTAT have lower mean power than burden and VT tests [24]. The finding of an association with burden and VT (unidirectional methods), but not with C-ALPHA and SKAT (bi-directional variance- component methods) are probably due to an excess of deleterious variants. Indeed, burden and VT tests are most powerful when the majority of rare variants have the same “unidirectional” effect (i.e., deleterious), whereas SKAT and C-ALPHA are mostly powered when both neutral and deleterious variants are present [18, 19, 24].

DVT patients carrying at least one rare *ADAMTS13* variant showed a reduction of ADAMTS13 activity levels, which was more pronounced considering variants predicted to be damaging (ß -25.9%, 95% CI -36.0, -16.0), in agreement with our previous study [8]. Conversely, controls carrying at least one rare *ADAMTS13* variant showed a slight and non-statistically significant reduction of ADAMTS13 activity levels compared with non-carriers. Unfortunately, none of the 5 controls carrying a rare potentially damaging variant with CADD > 20 had available plasma samples. As final step, to exclude that the association was biased by additional factors such as ADAMTS13 consumption during DVT event or drug administration, we repeated the analyses considering the 167 DVT cases whose plasma sample were collected ≥ 3 months from the acute event and/or not during anticoagulant therapy confirming our results (S3 Table).

The use of cumulative tests to assess the association between rare *VWF* variants and DVT did not show any association by considering all rare *VWF* variants or by restricting the analysis to potentially damaging variants. Only SKAT and SKAT-O showed a significant association after restriction to potentially damaging variants with CADD > 20. These results were unexpected as both methods should be optimal to test a mix of neutral and deleterious variants (i.e., before restriction analyses). However, the negative results obtained with the other methods, independently from the group of variants tested, led us to exclude an association between rare *VWF* variants and DVT. These findings, together with the absence of rare *F8* variants associated with DVT, led us to speculate that the increased VWF and FVIII levels measured in DVT patients could be due to other mechanisms such as the presence of mutations in regulatory regions or other genes involved in the VWF clearance [25–27].

Differently from our previous study, we identified 9 low-frequency/common variants potentially associated with DVT (P < 0.05) as the adjustment for multiple testing resulted in a high FDR (between 0.06 and 0.24). Of them, 7 were previously reported as *ADAMTS13* polymorphisms and showed a protective effect. This was partially explained considering that two groups of SNVs were in high linkage disequilibrium and showed the same association signal ([rs28641026, rs28503257; OR 0.53, 95% CI 0.31–0.89] and [rs3124768, rs3118667, rs739469; OR 0.78, 95% CI 0.67–0.92]). The effect of these SNVs was previously evaluated using *in vitro* expression studies (with the exception of the intronic variant rs739469) and none of them showed a severe reduction of ADAMTS13 activity levels [28, 29].

The remaining 2 SNVs localized in *VWF*, showed opposite effects and inconsistencies with previous reported studies. The rs1800382 showed the strongest association with DVT (OR 3.26, 95% CI 1.18–8.98), whereas it was previously described in VWD patients as responsible for a collagen type IV binding defect, hence resulting in a prolonged bleeding time instead of a thrombotic effect [30]. However, we cannot exclude that rs1800382 is in linkage disequilibrium with another *VWF* defect located in a region not covered by our study design, such as deep intronic or regulatory regions. The rs7962217 showed a protective effect. Nevertheless, its role is still uncertain because reported as associated with increased Factor VIII coagulant activity levels, but not with VWF antigen levels in a larger cohort of non-European Americans and African-Americans subjects [31]. Of the low-frequency/common variants identified, only three were located in *F8* and none of them was associated with DVT. This was in agreement with previous findings, demonstrating that increased FVIII levels were not due to the presence of common polymorphisms in *F8* [32].

This study could suffer from some limitations such as the relatively small sample size. However, the choice of striking criteria finalized to exclude the DVT patients who already had known genetic (e.g., FV Leiden and prothrombin G20210A mutations) or environmental risk factors for DVT, contributed to magnify the power. A second limitation could be related to the sequencing approach that was focused on the coding regions avoiding the identification of variants potentially located in the regulatory or intronic regions. Third, ADAMTS13 activity measurements were available for a part of the enrolled subjects. Finally, we cannot assume the generalizability of our findings in other different populations, as commonly reported in genetic studies.

In conclusion, rare *ADAMTS13* variants were associated with an increased risk for DVT. We confirmed that patients carrying a rare *ADAMTS13* variant had lower ADAMTS13 activity levels than non-carriers. These findings support the hypothesis that rare *ADAMTS13* variants may increase the DVT risk thru a mechanism that includes the reduction of ADAMTS13 activity. Conversely, 8 out of 9 top SNVs with low/common frequency showed a protective effect, although their association with DVT needs to be confirmed. Rare *VWF* and *F8* variants were not associated with DVT risk, suggesting that the increased plasma VWF and FVIII levels are caused by other mechanisms.

## Supporting information

S1 Table Common variants identified in Italian DVT patients and controls. (PDF)

S2 Table Rare variants identified in Italian DVT patients and controls. (PDF)

S3 Table Association between rare ADAMTS13 variants and ADAMTS13 activity levels in DVT patients whom blood sampling was performed not during treatment and at least three months after the DVT event. (PDF)

S4 Table DVT patients and controls carrying one out of 15 potentially damaging *ADAMTS13* SNVs with CADD>20. (PDF)
