# Supplementary material for: Role of ADAMTS13, VWF and F8 genes in deep vein thrombosis
Source: PLoS One. 2021 Oct 18;16(10):e0258675. doi: 10.1371/journal.pone.0258675 (PMC8523043; doi:10.1371/journal.pone.0258675)
Supplement: S1 Table — (PDF) [file pone.0258675.s001.pdf]

Table S1. Common variants identified in Italian DVT patients and controls

Chr	rs ID	Position	location	*Gene*	Ref. allele/ Risk allele	Protein Change	MAF (cases/controls)	OR (95%CI)[a]	P	FDR
9	rs3124768	136304497	exonic	*ADAMTS13*	G/A	p.T572T	0.43/0.50	0.78 (0.67-0.92)	0.003	0.06
9	rs739469	136298729	intronic	*ADAMTS13*	C/G	.	0.43/0.49	0.78 (0.67-0.92)	0.004	0.06
9	rs3118667	136291063	exonic	*ADAMTS13*	T/C	p.A140A	0.36/0.43	0.78 (0.66-0.92)	0.003	0.06
9	rs28641026	136314952	exonic	*ADAMTS13*	C/T	p.V970V	0.02/0.04	0.53 (0.31-0.89)	0.02	0.15
9	rs28503257	136319589	exonic	*ADAMTS13*	G/A	p.A1033T	0.02/0.04	0.53 (0.31-0.89)	0.02	0.15
9	rs3124767	136308542	exonic	*ADAMTS13*	T/C	p.G760G	0.45/0.50	0.82 (0.69-0.97)	0.02	0.15
9	rs685523	136310908	exonic	*ADAMTS13*	C/T	p.A900V	0.07/0.10	0.74 (0.55-0.99)	0.05	0.24
9	rs1055432	136324239	exonic	*ADAMTS13*	C/A	p.T1351T	0.24/0.28	0.84 (0.70-1.01)	0.06	0.31
9	rs34024143	136287582	exonic	*ADAMTS13*	C/T	p.R7W	0.09/0.11	0.85 (0.65-1.12)	0.26	0.81
9	rs2301612	136301982	exonic	*ADAMTS13*	C/G	p.Q448E	0.32/0.35	0.91 (0.77-1.08)	0.29	0.82
9	rs41314453	136307825	exonic	*ADAMTS13*	C/T	p.A732V	0.13/0.01	1.22 (0.57-2.64)	0.61	0.96
9	rs28647808	136305530	exonic	*ADAMTS13*	C/G	p.P618A	0.73/0.07	1.07 (0.79-1.44)	0.67	0.96
9	rs34934621	136319600	exonic	*ADAMTS13*	G/A	p.S1036S	0.5/0.05	1.10 (0.75-1.61)	0.62	0.96
9	rs36218903	136291286	intronic	*ADAMTS13*	G/A	.	0.02/0.02	0.88 (0.50-1.55)	0.65	0.96
9	rs36219245	136291469	intronic	*ADAMTS13*	T/G	.	0.06/0.06	1.07 (0.77-1.49)	0.67	0.96
9	rs34265876	136287207	UTR5	*ADAMTS13*	T/C	.	0.08/0.07	1.05 (0.77-1.43)	0.77	0.99
9	rs34054981	136291361	exonic	*ADAMTS13*	C/T	p.G194G	0.07/0.06	1.03 (0.75-1.43)	0.84	0.99
9	rs28571612	136290672	exonic	*ADAMTS13*	G/A	p.P118P	0.06/0.06	1.02 (0.73-1.43)	0.91	1.00
12	rs1800382	6128388	exonic	*VWF*	C/T	p.R1399H	0.01/0.004	3.26 (1.18-8.98)	0.02	0.15
12	rs7962217	6061559	exonic	*VWF*	C/T	p.G2705R	0.05/0.07	0.67 (0.47-0.95)	0.03	0.16
12	rs216311	6128443	exonic	*VWF*	C/T	p.T1381A	0.32/0.35	0.85 (0.72-1.01)	0.07	0.31
12	rs216867	6091000	exonic	*VWF*	G/A	p.T2413T	0.10/0.12	0.80 (0.62-1.03)	0.09	0.35
12	rs216868	6091164	intronic	*VWF*	G/A	.	0.28/0.31	0.86 (0.72-1.03)	0.10	0.38
12	rs216321	6143984	exonic	*VWF*	C/T	p.Q852R	0.06/0.07	0.80 (0.57-1.11)	0.18	0.61
12	rs35365059	6167118	exonic	*VWF*	C/T	p.A542A	0.06/0.07	0.84 (0.60-1.17)	0.29	0.82
12	rs216902	6105387	exonic	*VWF*	G/A	p.C1948C	0.36/0.38	0.92 (0.77-1.09)	0.31	0.83
12	rs35335161	6078424	exonic	*VWF*	A/T	p.F2561Y	0.05/0.28	0.84 (0.58-1.21)	0.34	0.88
12	rs56121649	6134949	intronic	*VWF*	C/G	.	0.25/0.28	0.89 (0.69-1.15)	0.38	0.90
12	rs71582881	6219916	intronic	*VWF*	C/G	.	0.02/0.01	1.35 (0.67-2.71)	0.40	0.90
12	rs1053523	6094784	exonic	*VWF*	T/C	p.T2282T	0.15/0.14	1.09 (0.86-1.38)	0.46	0.90
12	rs1800384	6127919	exonic	*VWF*	T/G	p.A1555A	0.08/0.06	1.13 (0.81-1.59)	0.47	0.90
12	rs1063857	6153514	exonic	*VWF*	A/G	p.Y795Y	0.36/0.37	0.94 (0.79-1.11)	0.45	0.90
12	rs1063856	6153534	exonic	*VWF*	T/C	p.T789A	0.36/0.37	0.94 (0.79-1.11)	0.46	0.90
12	rs61750615	6103650	exonic	*VWF*	G/A	p.P2063S	0.02/0.02	1.16 (0.62-2.20)	0.64	0.96
12	rs1800385	6127891	exonic	*VWF*	C/A	p.V1565L	0.09/0.08	1.08 (0.79-1.49)	0.63	0.96
12	rs1800383	6128170	exonic	*VWF*	C/G	p.D1472H	0.10/0.09	1.07 (0.81-1.42)	0.63	0.96
12	rs1800380	6138595	exonic	*VWF*	C/T	p.R960R	0.26/0.25	1.04 (0.86-1.25)	0.68	0.96
12	rs56068059	6134987	intronic	*VWF*	C/A	.	0.27/0.28	0.98 (0.77-1.24)	0.86	0.99
12	rs1800379	6167196	exonic	*VWF*	A/G	p.Y516Y	0.34/0.33	1.03 (0.86-1.23)	0.77	0.99
12	rs1800378	6172202	exonic	*VWF*	C/T	p.H484R	0.30/0.30	0.98 (0.82-1.17)	0.81	0.99
12	rs216305	6121078	intronic	*VWF*	T/C	.	0.04/0.43	1.02 (0.86-1.20)	0.85	0.99
12	rs1800387	6182828	exonic	*VWF*	A/T	p.N318K	0.04/0.04	1.04 (0.69-1.55)	0.87	0.99
12	rs1800377	6173433	exonic	*VWF*	C/T	p.V471I	0.09/0.09	0.97 (0.73-1.29)	0.84	0.98
12	rs1800376	6174414	exonic	*VWF*	T/G	p.S394S	0.13/0.13	1.01 (0.79-1.29)	0.93	1
12	rs1800375	6174423	exonic	*VWF*	T/A	p.T391T	0.13/0.13	1.00 (0.78-1.28)	0.97	1
23	rs1800292	154158201	exonic	*F8*	T/G	p.S1288S	0.10/0.10	NA	NA	1
23	rs1050705	154064200	UTR3	*F8*	T/C	.	0.23/0.22	NA	NA	1
23	rs1800291	154158285	exonic	*F8*	G/C	p.D1260E	0.12/0.12	NA	NA	1

Chr, chromosome; Ref. Allele, reference allele; MAF, Minor allele frequency; OR, Odds ratio; 95%CI, 95% confidence interval; P, P value; FDR, false discovery rate. [a]Age-, sex-adjusted.
