# Supplementary material for: Role of ADAMTS13, VWF and F8 genes in deep vein thrombosis
Source: PLoS One. 2021 Oct 18;16(10):e0258675. doi: 10.1371/journal.pone.0258675 (PMC8523043; doi:10.1371/journal.pone.0258675)
Supplement: S2 Table — (PDF) [file pone.0258675.s002.pdf]

Table S2. Rare variants identified in Italian DVT patients and controls

Chr	rs ID	Position	Location	Gene	Ref. allele/ Risk allele	Amino Acid Change	MAF Cases	MAF controls	MAF Study Population	1000 Genome Frequency	Potentially damaging [b]	CADD Score [c]
9	-	136287177	UTR5	*ADAMTS13*	G/A	-	0.001	0	0	0	0	3.522
9	rs36218240	136287396	UTR5	*ADAMTS13*	C/T	-	0.008	0.004	0.006	0.004	0	3.066
9	rs1036507778	136287418	UTR5	*ADAMTS13*	C/T	-	0.001	0.002	0.001	0	0	7.369
9	-	136287524	UTR5	*ADAMTS13*	C/T	-	0.001	0	0	0	0	8.036
9	-	136287530	UTR5	*ADAMTS13*	C/T	-	0	0.002	0.001	0	0	2.927
9	rs374861060	136287540	UTR5	*ADAMTS13*	C/T	-	0.001	0	0	0	0	6.087
9	rs77985067	136287578	exonic	*ADAMTS13*	C/T	p.H5H	0	0.001	0	0	0	0.743
9	rs201522226	136288251	exonic	*ADAMTS13*	G/A	p.V46M	0.001	0	0	0	1	14.53
9	-	136288278	exonic	*ADAMTS13*	C/T	p.P55S	0.001	0	0	0	1	0.258
9	rs782548827[a]	136289464	exonic	*ADAMTS13*	CAGAGG/-	p.Q72_R73del	0.001	0	0	0	1	10.16
9	rs782548827[a]	136289469	exonic	*ADAMTS13*	-/CAGAGG	p.R73_R74insQR	0	0.001	0	0	1	10.94
9	rs367627378	136289499	exonic	*ADAMTS13*	C/T	p.G77G	0.001	0	0	0	0	1.126
9	-	136289554	exonic	*ADAMTS13*	C/T	p.H96Y	0	0.001	0	0	1	25.2
9	rs142107133	136289598	exonic	*ADAMTS13*	C/T	p.I110I	0	0.002	0.001	0	0	13.34
9	rs147563206	136290675	exonic	*ADAMTS13*	C/T	p.S119S	0.004	0.002	0.003	0.002	0	10.75
9	rs782496629	136291054	intronic	*ADAMTS13*	G/A	-	0.001	0	0	0	0	4.044
9	rs781882283	136291058	exonic	*ADAMTS13*	G/T	p.G139C	0.001	0	0	0	1	22.6
9	-	136291263	intronic	*ADAMTS13*	C/T	-	0.001	0	0	0	0	0.609
9	rs199560983	136291275	intronic	*ADAMTS13*	C/T	-	0.001	0	0	0	0	5.996
9	rs148312697	136291338	exonic	*ADAMTS13*	G/C	p.D187H	0.001	0	0	0.001	1	24.4
9	-	136293832	exonic	*ADAMTS13*	G/A	p.A255A	0	0.003	0.002	0	0	12.53
9	-	136293839	exonic	*ADAMTS13*	G/A	p.A258T	0	0.003	0.002	0	1	13.72
9	-	136293844	exonic	*ADAMTS13*	C/T	p.G259G	0.01	0	0.002	0	0	14.3
9	-	136293846	exonic	*ADAMTS13*	T/C	p.L260P	0	0.003	0.002	0	1	16.79
9	-	136293847	exonic	*ADAMTS13*	C/A	p.L260L	0	0.003	0.002	0	0	14.16
9	-	136295071	exonic	*ADAMTS13*	G/A	p.A279A	0	0.001	0.001	0	0	12.38
9	rs36219562	136295170	exonic	*ADAMTS13*	C/T	p.R312R	0.001	0	0	0	0	9.727
9	-	136295198	exonic	*ADAMTS13*	T/C	p.C322R	0	0.001	0	0	1	24.1
9	rs781924046	136297805	exonic	*ADAMTS13*	G/A	p.V362M	0	0.001	0	0	1	6.442

Chr	rs ID	Position	Location	Gene	Ref. allele/ Risk allele	Amino Acid Change	MAF Cases	MAF controls	MAF Study Population	1000 Genome Frequency	Potentially damaging [b]	CADD Score [c]
9	rs145252342	136298513	exonic	*ADAMTS13*	C/T	p.R370C	0.001	0	0	0	1	23.4
9	-	136298519	exonic	*ADAMTS13*	C/G	p.R372G	0	0.001	0	0	1	17.12
9	rs151048660	136298562	exonic	*ADAMTS13*	G/A	p.R386H	0.001	0	0	0	1	12.63
9	rs142570561	136298605	exonic	*ADAMTS13*	C/T	p.C400C	0.001	0.002	0.001	0.004	0	13.54
9	rs36219902	136298758	intronic	*ADAMTS13*	C/T	-	0.001	0.003	0.002	0.001	0	16.51
9	rs145825553	136298777	exonic	*ADAMTS13*	C/T	p.R421C	0.005	0.001	0.003	0	1	25
9	rs36220239	136302008	exonic	*ADAMTS13*	G/T	p.Q456H	0.001	0	0	0	1	7.335
9	rs36220240	136302010	exonic	*ADAMTS13*	C/T	p.P457L	0.001	0	0	0.003	1	23.5
9	-	136302019	exonic	*ADAMTS13*	C/A	p.S460Y	0.001	0	0	0	1	15.1
9	rs11575933	136302063	exonic	*ADAMTS13*	C/T	p.P475S	0.001	0.001	0.001	0.001	1	9.965
9	-	136302934	exonic	*ADAMTS13*	A/C	p.S501R	0.001	0	0	0	1	15.43
9	rs148472763	136302984	exonic	*ADAMTS13*	G/C	p.G517G	0.003	0.004	0.003	0	0	6.309
9	-	136303005	exonic	*ADAMTS13*	G/A	p.S524S	0	0.001	0	0	0	12.43
9	rs374868673	136303482	exonic	*ADAMTS13*	G/A	p.A567A	0.001	0	0	0	0	0.915
9	rs36221216	136305475	exonic	*ADAMTS13*	C/T	p.I599I	0.001	0	0	0	0	0.038
9	rs34256013	136305488	exonic	*ADAMTS13*	G/A	p.V604I	0.001	0	0	0	1	4.04
9	rs36221217	136305508	exonic	*ADAMTS13*	C/T	p.S610S	0	0.001	0	0	0	1.44
9	-	136305520	exonic	*ADAMTS13*	C/T	p.N614N	0	0.001	0	0	0	4.523
9	rs371266006	136305529	exonic	*ADAMTS13*	C/T	p.Y617Y	0.001	0	0	0	0	6.322
9	rs782184721	136305609	exonic	*ADAMTS13*	G/A	p.R644H	0	0.002	0.001	0	1	0.003
9	rs199741568	136307537	exonic	*ADAMTS13*	C/T	p.G662G	0.001	0	0	0	0	0.087
9	rs149953167	136307560	exonic	*ADAMTS13*	G/A	p.R670H	0	0.001	0	0	1	10.66
9	-	136307577	exonic	*ADAMTS13*	A/C	p.T676P	0.001	0	0	0	1	18.88
9	rs36221451	136307848	exonic	*ADAMTS13*	G/A	p.E740K	0.001	0	0	0	1	6.099
9	rs781923426	136308530	exonic	*ADAMTS13*	C/T	p.A756A	0.001	0	0	0	0	1.904
9	-	136308583	exonic	*ADAMTS13*	G/T	p.G774V	0.001	0	0	0	1	22
9	-	136308639	exonic	*ADAMTS13*	G/C	p.A793P	0.001	0	0	0	1	10.15
9	-	136309985	exonic	*ADAMTS13*	T/C	p.W808R	0.001	0	0	0	1	5.517
9	rs782110632	136310048	exonic	*ADAMTS13*	G/A	p.E829K	0.001	0	0	0	1	3.398
9	rs36221472	136310071	exonic	*ADAMTS13*	T/C	p.D836D	0.001	0	0	0	1	0.279
9	rs140639242	136310108	exonic	*ADAMTS13*	G/A	p.V849I	0	0.002	0.001	0.001	1	0.278

Chr	rs ID	Position	Location	Gene	Ref. allele/ Risk allele	Amino Acid Change	MAF Cases	MAF controls	MAF Study Population	1000 Genome Frequency	Potentially damaging [b]	CADD Score [c]
9	rs147112200	136310143	exonic	*ADAMTS13*	C/T	p.V860V	0.001	0.002	0.001	0.001	0	0.156
9	rs782624187	136313715	intronic	*ADAMTS13*	C/T	-	0.002	0	0.001	0	0	0.025
9	-	136313742	exonic	*ADAMTS13*	G/C	p.L918L	0	0.001	0	0	0	8.696
9	rs782263547	136313761	exonic	*ADAMTS13*	A/G	p.R925G	0	0.002	0.001	0	1	6.427
9	-	136313812	exonic	*ADAMTS13*	C/T	p.R942W	0.001	0	0	0	1	23.6
9	rs782160285	136313816	exonic	*ADAMTS13*	G/A	p.R943Q	0.001	0	0	0	1	2.465
9	rs375151860	136313838	exonic	*ADAMTS13*	G/A	p.P950P	0.001	0	0	0	0	2.023
9	rs143568784	136313842	exonic	*ADAMTS13*	C/T	p.P952S	0.003	0.001	0.002	0.001	1	26.5
9	-	136313846	exonic	*ADAMTS13*	C/T	p.A953V	0.001	0	0	0	1	22.3
9	rs36222275	136314986	exonic	*ADAMTS13*	G/A	p.G982R	0.003	0	0.001	0.005	1	19.63
9	rs139808736	136315020	exonic	*ADAMTS13*	C/T	p.T993I	0	0.001	0	0	1	18.65
9	-	136315024	exonic	*ADAMTS13*	G/C	p.Q994H	0.001	0	0	0	1	22.8
9	rs36222579	136319642	exonic	*ADAMTS13*	G/A	p.V1050V	0	0.001	0	0	0	1.735
9	rs587731517	136319671	exonic	*ADAMTS13*	G/A	p.R1060Q	0	0.001	0	0.001	1	16.76
9	rs61751476	136320444	exonic	*ADAMTS13*	G/A	p.R1096H	0.003	0.002	0.002	0.001	1	0.263
9	rs281875303	136321665	exonic	*ADAMTS13*	G/T	p.G1183V	0.001	0	0	0	1	27.9
9	rs782197792	136321689	exonic	*ADAMTS13*	G/A	p.R1191Q	0	0.001	0	0	1	23.8
9	rs782767249	136321729	exonic	*ADAMTS13*	G/C	p.M1204I	0.001	0	0	0	1	9.581
9	rs371964138	136321769	exonic	*ADAMTS13*	C/T	p.R1218C	0.001	0	0	0	1	23.8
9	rs140450669	136321778	exonic	*ADAMTS13*	C/T	p.R1221W	0	0.001	0	0	1	6.435
9	rs36222899	136321795	exonic	*ADAMTS13*	G/A	p.V1226V	0.001	0	0	0	0	7.066
9	-	136321831	exonic	*ADAMTS13*	C/T	p.T1238T	0	0.001	0	0	0	1.454
9	rs782207691	136323069	exonic	*ADAMTS13*	C/T	p.I1254I	0.001	0	0	0	0	10.36
9	-	136323105	exonic	*ADAMTS13*	A/G	p.A1266A	0.001	0	0	0	0	10.13
9	rs377686931	136323156	exonic	*ADAMTS13*	C/T	p.I1283I	0	0.002	0.001	0	0	19.38
9	rs587756604	136323189	exonic	*ADAMTS13*	C/T	p.T1294T	0.001	0	0	0	0	2.852
9	-	136324269	exonic	*ADAMTS13*	C/T	p.D1361D	0.001	0	0	0	0	9.492
9	rs36223202	136324439	UTR3	*ADAMTS13*	T/C	-	0.001	0.001	0.001	0	0	6.026
9	rs142792179	136324470	UTR3	*ADAMTS13*	C/T	-	0.004	0.001	0.003	0.002	0	5.384
12	-	6058041	UTR3	*VWF*	C/A	-	0.001	0	0	0	0	7.492
12	-	6058123	UTR3	*VWF*	T/C	-	0	0.001	0	0	0	0.379

Chr	rs ID	Position	Location	Gene	Ref. allele/ Risk allele	Amino Acid Change	MAF Cases	MAF controls	MAF Study Population	1000 Genome Frequency	Potentially damaging [b]	CADD Score [c]
12	-	6058203	exonic	*VWF*	G/T	p.S2807Y	0.001	0	0	0	1	14.69
12	rs371036946	6058257	exonic	*VWF*	G/C	p.T2789S	0	0.001	0	0	1	18.84
12	rs78353028	6061675	exonic	*VWF*	G/A	p.T2666M	0.002	0.001	0.001	0	1	10.65
12	rs149834874	6061684	exonic	*VWF*	C/G	p.R2663P	0.008	0.003	0.005	0.003	1	16.98
12	-	6062697	exonic	*VWF*	T/C	p.I2651V	0.001	0	0	0	1	22.6
12	rs61751302	6062708	exonic	*VWF*	G/A	p.T2647M	0.003	0.001	0.002	0.007	1	3.822
12	rs201953086	6062743	exonic	*VWF*	A/C	p.N2635K	0.001	0	0	0	1	7.641
12	rs147052620	6076728	exonic	*VWF*	C/T	p.R2604H	0.001	0	0	0	1	0.045
12	rs754245049	6077285	intronic	*VWF*	C/T	-	0	0.001	0	0	0	6.265
12	rs137987906	6078502	exonic	*VWF*	C/T	p.R2535Q	0	0.001	0	0	1	23
12	rs562305031	6078534	exonic	*VWF*	C/T	p.P2524P	0.001	0	0	0	0	0.063
12	rs374690023	6078535	exonic	*VWF*	G/A	p.P2524L	0	0.001	0	0	1	13.19
12	rs111752224	6085281	exonic	*VWF*	C/T	p.R2478Q	0	0.001	0	0	1	3.193
12	-	6085331	exonic	*VWF*	C/T	p.M2461I	0	0.001	0	0	1	22
12	rs55944252	6085370	exonic	*VWF*	G/A	p.C2448C	0	0.001	0	0.002	0	2.796
12	rs151303589	6090958	exonic	*VWF*	G/A	p.P2427P	0.001	0	0	0	0	6.842
12	-	6091152	exonic	*VWF*	T/C	p.R2363G	0	0.001	0	0	1	24.2
12	rs779492794	6092340	exonic	*VWF*	C/T	p.E2353K	0	0.001	0	0	1	26.3
12	-	6092341	exonic	*VWF*	G/T	p.G2352G	0	0.001	0	0	0	9.071
12	rs749720005	6092365	exonic	*VWF*	G/A	p.L2344L	0	0.001	0	0	0	7.014
12	-	6092375	exonic	*VWF*	T/G	p.E2341A	0.001	0	0	0	1	17.66
12	rs201792015	6094222	exonic	*VWF*	T/A	p.E2322V	0	0.001	0	0	1	22.8
12	rs267607357	6094255	exonic	*VWF*	C/T	p.R2311H	0	0.001	0	0	1	15.2
12	rs371531354	6094278	exonic	*VWF*	C/T	p.T2303T	0.001	0.001	0.001	0	0	0.004
12	rs112046757	6094290	intronic	*VWF*	A/T	-	0.001	0	0	0	0	4.694
12	rs752321350	6094291	intronic	*VWF*	G/A	-	0	0.001	0	0	0	3.161
12	rs61750625	6094771	exonic	*VWF*	G/A	p.R2287W	0	0.001	0	0	1	25.9
12	rs71581020	6101027	exonic	*VWF*	C/T	p.E2252E	0.002	0.002	0.002	0.001	0	9.9
12	rs748791680	6101065	exonic	*VWF*	C/G	p.D2240H	0.001	0	0	0	1	0.07
12	-	6103064	exonic	*VWF*	C/T	p.G2188R	0.001	0	0	0	1	25.4
12	rs2229446	6103072	exonic	*VWF*	C/T	p.R2185Q	0.005	0.004	0.004	0.002	1	27.2

Chr	rs ID	Position	Location	Gene	Ref. allele/ Risk allele	Amino Acid Change	MAF Cases	MAF controls	MAF Study Population	1000 Genome Frequency	Potentially damaging [b]	CADD Score [c]
12	rs34230288	6103094	exonic	*VWF*	C/A	p.A2178S	0.009	0.007	0.009	0.022	1	8.066
12	rs200719767	6103274	exonic	*VWF*	G/A	p.R2118W	0	0.001	0	0	1	22.7
12	rs11537642	6103281	exonic	*VWF*	A/T	p.T2115T	0.002	0.001	0.001	0.001	0	0.368
12	rs115914543	6103323	exonic	*VWF*	G/T	p.G2101G	0	0.001	0	0.001	0	3.645
12	rs769985279	6103416	intronic	*VWF*	G/A	-	0.001	0.001	0.001	0	0	0.688
12	rs752835830	6103725	exonic	*VWF*	T/C	p.M2038V	0	0.001	0	0	1	3.61
12	rs55784921	6103738	exonic	*VWF*	G/A	p.Y2033Y	0.002	0.006	0.004	0.002	0	0.073
12	-	6105180	exonic	*VWF*	G/T	p.H2017Q	0.001	0	0	0	1	15.13
12	rs34444862	6105396	intronic	*VWF*	G/C	-	0.009	0.005	0.007	0.022	0	3.455
12	-	6120797	exonic	*VWF*	C/T	p.R1943H	0.001	0.001	0.001	0	1	23.1
12	rs55804572	6121125	intronic	*VWF*	C/A	-	0.003	0.002	0.003	0.003	0	3.213
12	rs58238683	6121147	intronic	*VWF*	A/G	-	0.003	0.002	0.003	0.003	0	7.979
12	-	6121182	intronic	*VWF*	T/C	-	0.001	0	0	0	0	11.74
12	-	6121198	intronic	*VWF*	A/T	-	0	0.001	0	0	0	9.653
12	rs55664929	6121220	intronic	*VWF*	C/T	-	0.003	0.002	0.003	0	0	0.718
12	rs7961539	6121238	intronic	*VWF*	C/T	-	0.002	0.001	0.001	0	0	6.88
12	rs367952240	6125307	exonic	*VWF*	C/T	p.T1801T	0.001	0	0	0	0	0.277
12	rs2229448	6125397	exonic	*VWF*	C/A	p.G1771G	0.005	0.001	0.003	0.001	0	6.322
12	rs200237834	6125417	intronic	*VWF*	T/G	-	0	0.002	0.001	0.002	0	1.955
12	rs780724104	6125421	intronic	*VWF*	G/A	-	0	0.001	0	0	0	1.39
12	rs375741263	6125422	intronic	*VWF*	C/T	-	0	0.001	0	0	0	2.481
12	rs759461354	6125450	intronic	*VWF*	C/T	-	0.001	0.001	0.001	0	0	0.136
12	rs539229468	6125461	intronic	*VWF*	C/T	-	0.002	0	0.001	0	0	0.004
12	-	6125462	intronic	*VWF*	G/A	-	0.002	0	0.001	0	0	0.499
12	rs765280599	6125491	intronic	*VWF*	A/G	-	0	0.001	0	0	0	1.933
12	rs769299722	6125676	intronic	*VWF*	C/T	-	0.001	0	0	0	0	11.61
12	rs199729586	6125708	exonic	*VWF*	T/C	p.Q1762R	0.001	0	0	0	1	22.2
12	rs61750604	6125715	exonic	*VWF*	C/T	p.V1760I	0.001	0.001	0.001	0.003	1	2.544
12	rs41276736	6125716	exonic	*VWF*	G/A	p.D1759D	0.006	0.003	0.004	0.012	0	2.227
12	rs61750603	6125802	exonic	*VWF*	A/T	p.S1731T	0.004	0.002	0.003	0.001	1	23.1
12	rs78302129	6125820	exonic	*VWF*	G/A	p.P1725S	0.001	0	0	0	1	18.74

Chr	rs ID	Position	Location	Gene	Ref. allele/ Risk allele	Amino Acid Change	MAF Cases	MAF controls	MAF Study Population	1000 Genome Frequency	Potentially damaging [b]	CADD Score [c]
12	rs11063986	6125909	intronic	*VWF*	C/G	-	0	0.001	0	0	0	0.057
12	rs61750601	6125910	intronic	*VWF*	G/A	-	0.008	0.004	0.006	0.0089	0	0.047
12	rs765667845	6126023	exonic	*VWF*	G/C	p.P1689P	0.001	0	0	0	0	11.33
12	-	6127537	exonic	*VWF*	C/G	p.A1683P	0	0.001	0	0	1	9.999
12	rs767567453	6127561	exonic	*VWF*	G/A	p.L1675L	0.001	0	0	0	0	0.997
12	rs1800386	6127833	exonic	*VWF*	T/C	p.Y1584C	0.004	0.005	0.005	0.004	1	24.3
12	rs769921684	6128047	exonic	*VWF*	C/T	p.D1513N	0.001	0	0	0	1	13.31
12	rs774879567	6128080	exonic	*VWF*	C/T	p.V1502I	0	0.001	0	0	1	14.91
12	rs138966048	6128126	exonic	*VWF*	C/T	p.S1486S	0.001	0	0	0	0	0.015
12	rs149424724	6128127	exonic	*VWF*	G/A	p.S1486L	0.001	0.002	0.001	0	1	12.4
12	rs777683033	6128132	exonic	*VWF*	C/T	p.G1484G	0.001	0.001	0.001	0	0	0.615
12	rs144796763	6128141	exonic	*VWF*	C/A	p.G1481G	0.002	0.002	0.002	0.001	0	0.363
12	-	6128267	exonic	*VWF*	C/G	p.V1439V	0	0.001	0	0	0	3.39
12	rs11063987	6128280	exonic	*VWF*	T/C	p.N1435S	0.001	0.001	0.001	0	1	21.6
12	-	6128294	exonic	*VWF*	C/A	p.K1430N	0	0.001	0	0	1	20.3
12	-	6128333	exonic	*VWF*	C/-	p.H1419Mfsa21	0.001	0	0	0	1	32
12	rs368639720	6128378	exonic	*VWF*	C/T	p.Q1402Q	0.001	0	0	0	0	7.374
12	rs1800382	6128388	exonic	*VWF*	C/T	p.R1399H	0.014	0.004	0.009	0.004	1	23
12	rs140464171	6128438	exonic	*VWF*	C/A	p.L1382L	0.002	0.001	0.001	0.002	0	5.247
12	rs762377320	6128444	exonic	*VWF*	G/A	p.I1380I	0	0.001	0	0	0	0.801
12	rs11063988	6128446	exonic	*VWF*	T/C	p.I1380V	0.001	0.001	0.001	0	1	1.747
12	rs61750070	6128469	exonic	*VWF*	A/C	p.I1372S	0.001	0	0	0	1	16.01
12	rs1800381	6128573	exonic	*VWF*	C/T	p.P1337P	0.001	0.001	0.001	0	0	0.667
12	rs61749370[a]	6128787	exonic	*VWF*	G/A	p.P1266L	0.001	0.005	0.003	0	1	22.9
12	rs61749370[a]	6128787	exonic	*VWF*	G/T	p.P1266Q	0.001	0.005	0.003	0.001	1	22.9
12	rs2228319	6128789	exonic	*VWF*	C/T	p.P1265P	0.003	0.001	0.002	0	0	0.558
12	rs199831474	6128795	exonic	*VWF*	C/T	p.S1263S	0.004	0.011	0.008	0	0	0.021
12	rs150576611	6128865	exonic	*VWF*	G/A	p.P1240L	0	0.001	0	0.001	1	16.82
12	rs61749368[a]	6128892	exonic	*VWF*	T/G	p.N1231T	0	0.001	0	0.001	1	16.95
12	rs61749368[a]	6128892	exonic	*VWF*	T/C	p.N1231S	0	0.001	0	0	1	16.95
12	rs61749367	6128898	exonic	*VWF*	A/C	p.V1229G	0.003	0.003	0.003	0.001	1	19.8

Chr	rs ID	Position	Location	Gene	Ref. allele/ Risk allele	Amino Acid Change	MAF Cases	MAF controls	MAF Study Population	1000 Genome Frequency	Potentially damaging [b]	CADD Score [c]
12	-	6131070	exonic	*VWF*	T/G	p.I1224L	0.001	0.001	0.001	0	1	19.44
12	-	6131077	exonic	*VWF*	G/C	p.H1221Q	0.001	0.001	0.001	0	1	20.4
12	rs16933969	6131161	exonic	*VWF*	A/G	p.P1193P	0.002	0.001	0.001	0	0	0.83
12	rs113446850	6132790	intronic	*VWF*	T/G	-	0.002	0.001	0.001	0	0	7.746
12	rs139579968	6132797	exonic	*VWF*	G/A	p.P1127S	0	0.001	0	0	1	28.4
12	rs118072723	6134965	intronic	*VWF*	G/A	-	0.002	0	0.001	0	0	3.851
12	-	6134983	intronic	*VWF*	C/A	-	0	0.002	0.001	0	0	0.81
12	-	6135025	intronic	*VWF*	C/G	-	0.001	0	0.001	0	0	8.131
12	rs200293808	6135068	intronic	*VWF*	G/A	-	0	0.002	0.001	0	0	2.747
12	rs368366214	6135077	exonic	*VWF*	TGG/-	p.T1034del	0	0.001	0	0	1	20.2
12	-	6135218	intronic	*VWF*	G/A	-	0	0.001	0	0	0	0.018
12	rs376548659	6138531	exonic	*VWF*	C/T	p.V982M	0	0.002	0.001	0	1	24
12	rs150418484	6138561	exonic	*VWF*	C/T	p.V972M	0.001	0	0	0	1	20.9
12	rs33978901	6140659	exonic	*VWF*	C/T	p.R924Q	0.008	0.008	0.008	0.023	1	21.7
12	rs35191786	6140691	exonic	*VWF*	T/G	p.G913G	0	0.001	0	0	0	5.498
12	rs753317915	6143866	exonic	*VWF*	G/A	p.Y891Y	0.001	0	0	0	0	7.798
12	rs34510401	6143953	exonic	*VWF*	C/A	p.V862V	0.003	0.003	0.003	0.002	0	9.342
12	rs41276738	6143978	exonic	*VWF*	C/T	p.R854Q	0.003	0.003	0.003	0.004	1	33
12	rs75645183	6145590	exonic	*VWF*	G/T	p.A837D	0.001	0	0	0	1	17
12	rs151048762	6145603	exonic	*VWF*	C/T	p.G833S	0.001	0	0	0.001	1	26.1
12	rs62643631	6153464	exonic	*VWF*	G/A	p.P812L	0	0.001	0	0.002	1	20.8
12	rs771423537	6153513	exonic	*VWF*	C/T	p.D796N	0	0.001	0	0	1	28.6
12	rs61748472	6153554	exonic	*VWF*	C/T	p.R782Q	0.001	0	0	0	1	10.99
12	rs2228317	6155950	exonic	*VWF*	C/T	p.M740I	0.003	0	0.001	0	1	11.75
12	rs377672051	6161797	exonic	*VWF*	C/G	p.D700H	0.001	0	0	0	1	9.197
12	rs745612376	6161802	exonic	*VWF*	C/T	p.R698K	0	0.002	0.001	0	1	3.965
12	rs61754019	6166046	exonic	*VWF*	G/A	p.A641V	0	0.003	0.002	0	1	14.62
12	rs267607308	6166187	exonic	*VWF*	G/C	p.A594G	0.002	0	0.001	0	1	25.6
12	rs569984866	6166243	intronic	*VWF*	G/A	-	0.001	0	0	0	0	11.37
12	rs141649383	6167119	exonic	*VWF*	G/C	p.A542G	0.002	0.001	0.001	0.001	1	18.39
12	rs138268387	6167130	exonic	*VWF*	G/A	p.P538P	0	0.001	0	0.001	0	2.645

Chr	rs ID	Position	Location	Gene	Ref. allele/ Risk allele	Amino Acid Change	MAF Cases	MAF controls	MAF Study Population	1000 Genome Frequency	Potentially damaging [b]	CADD Score [c]
12	-	6172159	exonic	*VWF*	C/T	p.L498L	0	0.001	0	0	0	14.53
12	rs761973219	6172220	exonic	*VWF*	C/T	p.G478D	0	0.001	0	0	1	25.2
12	rs66722092	6172230	intronic	*VWF*	A/G	-	0	0.001	0	0	0	5.12
12	rs61754007	6174316	exonic	*VWF*	A/G	p.I427T	0	0.001	0	0	1	26.5
12	rs745805859	6181508	exonic	*VWF*	G/A	p.D366D	0	0.001	0	0	0	12.26
12	rs71582884	6181529	exonic	*VWF*	G/A	p.P359P	0.003	0.002	0.002	0.004	0	0.062
12	-	6204679	exonic	*VWF*	G/A	p.R202W	0.001	0	0	0	1	24.4
12	rs143054357	6204737	exonic	*VWF*	C/T	p.S182S	0.003	0.001	0.002	0.001	0	2.782
12	rs779098641	6219599	exonic	*VWF*	G/A	p.T158I	0	0.001	0	0	1	27.7
12	rs76505074	6219681	exonic	*VWF*	C/T	p.G131S	0.003	0.003	0.003	0.001	1	15.74
12	rs2229444	6219682	exonic	*VWF*	G/A	p.S130S	0.005	0.007	0.006	0.005	0	9.971
12	rs753526249	6219797	intronic	*VWF*	G/T	-	0.001	0	0	0	0	0.956
12	rs150411483	6219808	intronic	*VWF*	C/T	-	0.003	0.004	0.003	0.008	0	4.631
12	-	6219840	intronic	*VWF*	T/C	-	0	0.001	0	0	0	2.585
12	-	6219902	intronic	*VWF*	C/A	-	0.001	0	0	0	0	1.958
12	rs777859406	6220134	exonic	*VWF*	C/G	p.G74A	0.001	0	0	0	1	25.1
12	rs200901782	6230399	exonic	*VWF*	G/A	p.A54V	0	0.001	0	0	1	15.99
12	-	6230416	exonic	*VWF*	C/T	p.G48G	0	0.001	0	0	0	10.28
12	rs2229443	6230446	exonic	*VWF*	G/A	p.F38F	0.001	0	0	0.003	0	7.477
12	rs537944350	6230465	exonic	*VWF*	G/A	p.T32M	0.001	0	0	0	1	0.164
12	rs770031169	6230500	exonic	*VWF*	G/C	p.T20T	0.001	0	0	0	0	1.091
12	rs114713980	6232300	intronic	*VWF*	G/T	-	0	0.001	0	0.001	0	9.84
12	-	6233689	UTR5	*VWF*	A/G	-	0	0.001	0	0	0	5.401
X	-	154064256	UTR3	*F8*	C/T	-	0	0.002	0.001	0	0	6.978
X	rs36101366	154064493	UTR3	*F8*	T/C	-	0.008	0.006	0.007	0.001	0	11.52
X	rs782733383	154064567	UTR3	*F8*	T/C	-	0.002	0	0.001	0.001	0	9.611
X	rs1396947	154064580	UTR3	*F8*	T/C	-	0	0.001	0	0.76	0	6.903
X	rs192048301	154064812	UTR3	*F8*	G/A	-	0.002	0	0.001	0.001	0	4.296
X	-	154065160	UTR3	*F8*	T/C	-	0	0.001	0	0	0	2.794
X	-	154065209	UTR3	*F8*	C/T	-	0	0.002	0.001	0	0	4.142
X	rs782435894	154065794	UTR3	*F8*	G/A	-	0.001	0.001	0.001	0	0	7.048

Chr	rs ID	Position	Location	Gene	Ref. allele/ Risk allele	Amino Acid Change	MAF Cases	MAF controls	MAF Study Population	1000 Genome Frequency	Potentially damaging [b]	CADD Score [c]
X	rs781949424	154065905	exonic	*F8*	C/T	p.E2341E	0.001	0	0	0	0	8.825
X	rs137852474	154065960	exonic	*F8*	C/T	p.R2323H	0.001	0	0	0	1	22.5
X	rs1800297	154088838	exonic	*F8*	T/C	p.M2257V	0.002	0	0.001	0.001	1	17.33
X	rs782217392	154091435	exonic	*F8*	C/T	p.R2166Q	0	0.002	0.001	0	1	14.82
X	-	154130393	exonic	*F8*	C/A	p.R2016R	0	0.002	0.001	0	0	8.685
X	rs782722177	154132195	exonic	*F8*	T/C	p.Y1995C	0.001	0	0	0	1	24
X	rs137852363	154132301	exonic	*F8*	G/T	p.R1960R	0	0.001	0	0	0	15.49
X	rs370451061	154132405	intronic	*F8*	A/G	-	0.001	0	0	0	0	0.959
X	-	154133051	intronic	*F8*	C/A	-	0.001	0	0	0	0	2.637
X	-	154133155	exonic	*F8*	T/C	p.Q1839Q	0.001	0	0	0	0	12.9
X	rs782235155	154157002	exonic	*F8*	G/A	p.S1688L	0.001	0	0	0	1	15.18
X	rs781987290	154157219	exonic	*F8*	T/C	p.T1616A	0	0.001	0	0	1	6.137
X	-	154157812	exonic	*F8*	A/G	p.I1418T	0	0.002	0.001	0	1	6.073
X	rs183178693	154157961	exonic	*F8*	C/T	p.P1368P	0	0.001	0	0	0	0.149
X	rs140160067	154158132	exonic	*F8*	T/C	p.A1311A	0	0.001	0	0	0	1.693
X	rs782019600	154159104	exonic	*F8*	C/G	p.E987D	0	0.001	0	0	1	8.225
X	-	154159267	exonic	*F8*	T/C	p.D933G	0	0.002	0.001	0	1	5.245
X	rs782201289	154159737	exonic	*F8*	A/G	p.N776N	0	0.001	0	0	0	0.288
X	-	154159914	exonic	*F8*	C/A	p.R717R	0.001	0	0	0	0	10.33
X	rs35383156	154189379	exonic	*F8*	C/T	p.R503H	0	0.002	0.001	0	1	23.9
X	-	154194465	intronic	*F8*	A/G	-	0.001	0	0	0	0	9.925
X	rs145623784	154194709	exonic	*F8*	G/A	p.P421P	0.002	0	0.001	0	0	0.86
X	-	154194919	exonic	*F8*	T/C	p.E351E	0	0.001	0	0	0	9.336
X	rs35621875	154221432	intronic	*F8*	G/A	-	0.002	0	0.001	0.001	0	3.534
X	-	154227832	exonic	*F8*	C/T	p.V63I	0	0.001	0	0	1	8.712
X	rs1800283	154250726	exonic	*F8*	G/A	p.D34D	0.002	0.002	0.002	0	0	12.96

MAF, risk allele frequency; [a] Tri-allelic variants; [b] Rare variants classified as potentially damaging (missense mutations, frameshift mutations, deletions and insertions [1]), whereas the other variants were considered as not damaging [0]. [c] Potentially damaging variants [1] with a CADD Score > 20.
