# Supplementary material for: Role of ADAMTS13, VWF and F8 genes in deep vein thrombosis
Source: PLoS One. 2021 Oct 18;16(10):e0258675. doi: 10.1371/journal.pone.0258675 (PMC8523043; doi:10.1371/journal.pone.0258675)
Supplement: S3 Table — (PDF) [file pone.0258675.s003.pdf]

Table S3. Association between rare *ADAMTS13* variants and ADAMTS13 activity levels in DVT patients whom blood sampling was performed not during treatment and at least three months after the DVT event.

	Carriers		Non-carriers		Linear Regression [a]	
**Variant Class**	**All, n**	**ADAMTS13 Activity (%)**	**All, n**	**ADAMTS13 Activity (%)**	**ß (95% CI)**	**P**
All rare	52	93 (81-106)	407	97 (85-111)	-6.9 (-12.0, -1.4)	0.015
Potentially damaging [b]	22	90 (72-110)	437	97 (85-110)	-9.0 (-17.0, 0.76)	<0.001
CADD > 20	7	71 (54-85)	452	97 (85-111)	-28.0 (-42.0, -14)	<0.001
**Variant Class**	**Cases, n**	**ADAMTS13 Activity (%)**	**Cases, n**	**ADAMTS13 Activity (%)**	**ß (95% CI)**	**P**
All rare	24	90 (72-96)	143	95 (82-109)	-10.0 (-18.0, -1.8)	0.017
Potentially damaging [b]	13	81 (60-95)	154	95 (82-108)	-14.0 (-25.0, -3.6)	0.009
CADD > 20	7	71 (54-85)	160	95 (82-108)	-24.0 (-38.0, -9.8)	0.001
**Variant Class**	**Controls, n**	**ADAMTS13 Activity (%)**	**Controls, n**	**ADAMTS13 Activity (%)**	**ß (95% CI)**	**P**
All rare	28	98 (85-112)	264	98 (86-111)	-2.9 (-10.4, 4.5)	0.4
Potentially damaging [b]	9	100 (86-114)	283	98 (86-111)	-2.4 (-10.0, 15.0)	0.7
CADD > 20	0	-	292	98 (86-111)	-	

Analyses have been performed by considering at first, all carriers of at least one rare *ADAMTS13* variant and then by dividing carriers in DVT cases and controls. ADAMTS13 activity levels (expressed as percentage) was reported as median and interquartile range. Linear regression results were reported as beta coefficients (ß) and 95% confidence intervals (CI). [a] The model was age and sex adjusted. [b] Rare variants classified as potentially damaging (missense mutations, frameshift mutations, deletions and insertions).
