# Supplementary material for: Role of ADAMTS13, VWF and F8 genes in deep vein thrombosis
Source: PLoS One. 2021 Oct 18;16(10):e0258675. doi: 10.1371/journal.pone.0258675 (PMC8523043; doi:10.1371/journal.pone.0258675)
Supplement: S4 Table — (PDF) [file pone.0258675.s004.pdf]

S4 Table. DVT patients and controls carrying one out of 15 potentially damaging *ADAMTS13* SNVs with CADD > 20

rs ID	Position	Case/Control	Amino Acid Change	Domain	MAF Cases	MAF Controls	CADD [a]	ADAMTS13 Activity (%)
**rs148312697**	136291338	Case	p.D187H	MP	0.001	0	24.4	58
**rs145825553**	136298777	Case	p.R421C [b]	TSP-1	0.005	0.001	25	57
		Case						76
		Case						63
		Case						71
		Case						49
		Case						-
		Control						-
**rs36220240**	136302010	Case	p.P457L	CYS	0.001	0	23.5	89
**-**	136308583	Case	p.G774V	TSP-3	0.001	0	22	60
**-**	136313812	Case	p.R942W	TSP-5	0.001	0	23.6	90
**rs143568784**	136313842	Case	p.P952S	TSP-6	0.003	0.001	26.5	58
		Case						95
		Case						-
		Control						-
**-**	136313846	Case	p.A953V	TSP-6	0.001	0	22.3	81
**rs371964138**	136321769	Case	p.R1218C	CUB-1	0.001	0	23.8	42
**-**	136295198	Control	p.C322R	DIS	0	0.001	24.1	-
**rs782197792**	136321689	Control	p.R1191Q	TSP-8	0	0.001	23.8	-
**-**	136289554	Control	p.H96Y	MP	0	0.001	25.2	-
**rs281875303**	136321665	Case	p.G1183V	TSP-8	0.001	0	27.9	-
**rs145252342**	136298513	Case	p.R370C	DIS	0.001	0	23.4	-
**-**	136315024	Case	p.Q994H	TSP-6	0.001	0	22.8	-
**rs781882283**	136291058	Case	p.G139C	MP	0.001	0	22.6	-

Sex: M, Male; F, Female; MAF, Minor allele frequency. [a] Variants with a CADD Score > 20 were considered as damaging.

[b] 2 cases and 1 control have been reported in the frame of a previous study (Pagliari et al; Plos One 2016).
